# Nutritional Aspects of Ecologically Relevant Phytochemicals in Ruminant Production

**DOI:** 10.3389/fvets.2021.628445

**Published:** 2021-03-05

**Authors:** Luis O. Tedeschi, James P. Muir, Harley D. Naumann, Aaron B. Norris, Carlos A. Ramírez-Restrepo, Susanne U. Mertens-Talcott

**Affiliations:** ^1^Department of Animal Science, Texas A&M University, College Station, TX, United States; ^2^Texas A&M AgriLife Research, Stephenville, TX, United States; ^3^Division of Plant Sciences, University of Missouri, Columbia, MO, United States; ^4^Department of Natural Resources Management, Texas Tech University, Lubbock, TX, United States; ^5^CR Eco-efficient Agriculture Consultancy (CREAC)™, Bushland Beach, QLD, Australia; ^6^Department of Nutrition and Food Science, Texas A&M University, College Station, TX, United States

**Keywords:** feed additive, methods, nutrition, rumen modifiers, ruminants, antinutritive factor

## Abstract

This review provides an update of ecologically relevant phytochemicals for ruminant production, focusing on their contribution to advancing nutrition. Phytochemicals embody a broad spectrum of chemical components that influence resource competence and biological advantage in determining plant species' distribution and density in different ecosystems. These natural compounds also often act as plant defensive chemicals against predatorial microbes, insects, and herbivores. They may modulate or exacerbate microbial transactions in the gastrointestinal tract and physiological responses in ruminant microbiomes. To harness their production-enhancing characteristics, phytochemicals have been actively researched as feed additives to manipulate ruminal fermentation and establish other phytochemoprophylactic (prevent animal diseases) and phytochemotherapeutic (treat animal diseases) roles. However, phytochemical-host interactions, the exact mechanism of action, and their effects require more profound elucidation to provide definitive recommendations for ruminant production. The majority of phytochemicals of nutritional and pharmacological interest are typically classified as flavonoids (9%), terpenoids (55%), and alkaloids (36%). Within flavonoids, polyphenolics (e.g., hydrolyzable and condensed tannins) have many benefits to ruminants, including reducing methane (CH_4_) emission, gastrointestinal nematode parasitism, and ruminal proteolysis. Within terpenoids, saponins and essential oils also mitigate CH_4_ emission, but triterpenoid saponins have rich biochemical structures with many clinical benefits in humans. The anti-methanogenic property in ruminants is variable because of the simultaneous targeting of several physiological pathways. This may explain saponin-containing forages' relative safety for long-term use and describe associated molecular interactions on all ruminant metabolism phases. Alkaloids are N-containing compounds with vast pharmacological properties currently used to treat humans, but their phytochemical usage as feed additives in ruminants has yet to be exploited as they may act as ghost compounds alongside other phytochemicals of known importance. We discussed strategic recommendations for phytochemicals to support sustainable ruminant production, such as replacements for antibiotics and anthelmintics. Topics that merit further examination are discussed and include the role of fresh forages vis-à-vis processed feeds in confined ruminant operations. Applications and benefits of phytochemicals to humankind are yet to be fully understood or utilized. Scientific explorations have provided promising results, pending thorough vetting before primetime use, such that academic and commercial interests in the technology are fully adopted.

## Introduction

Ideal anaerobic fermentation in the rumen relies on a steady supply of substrate (i.e., quantity and frequency), preservation of a favorable condition for microbial growth (e.g., temperature, pH, substrate mixing), and constant removal of undesirable substances (e.g., bacterial toxins, hydrogen), so that the profile and amount of volatile fatty acids (VFA) produced and microbial protein leaving the rumen meets the ruminant's daily requirements for energy and protein without having deleterious impacts in the rumen health and functionality (e.g., rumenitis) (1, 2). Although the rumen can function adequately if these conditions are met, it may not be operating at its maximum anaerobic efficiency. Thus, some dietary tweaking might achieve maximum anaerobic efficiency or maintain a healthy operational rumen.

This is where feed additives, also known as rumen modifiers, come into play. If the feed additive is of plant origin, i.e., phytogenic, they are collectively referred to as phytochemicals. Some usually refer to them as plant secondary metabolites because they are not associated with essential roles in the plant, such as photosynthesis, respiration, and growth and development (3). However, the distinction between primary and secondary metabolites is obscure and relative to the plant's physiological needs. For instance, environmental conditions and ecological niches drive the synthesis of different phenolics that are entrenched in the plant's genome based on their evolutionary strategies, but the reasons for evolutionary demands, however, are unclear (4).

Phytochemicals of nutritional and pharmacological interest, such as those to prevent (phytochemoprophylaxis) or treat (phytochemotherapeutic) animal diseases, are typically classified as flavonoids (e.g., polyphenolics), terpenoids (e.g., terpenes), and alkaloids (3). Not all phytochemicals have known beneficial properties to ruminants, but those that do so are often grouped as polyphenolics (e.g., hydrolyzable—HT—and condensed—CT—tannins), terpenes (e.g., saponins), vitamins, and essential oils (EO). In part, the immense variability in phytochemical biological properties makes it very difficult to catalog them and study their effects on animals of economic relevance.

**Figure 1:**
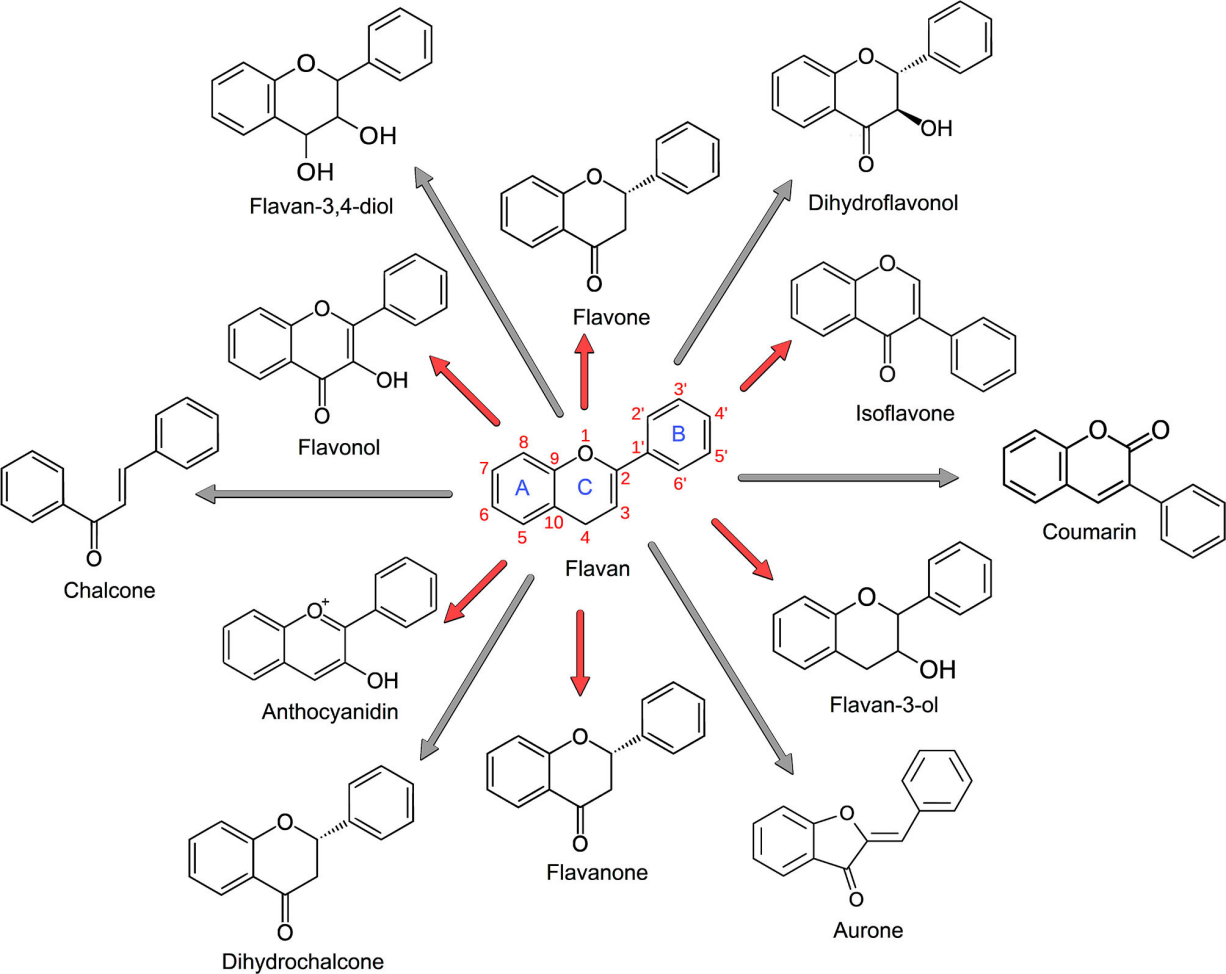
Flavonoid nomenclature. The main subclasses of flavonoids are shown in red arrows, including anthocyanidins, flavan-3-ol, flavanones, flavones, flavonols, and isoflavones. The minor flavonoids are shown in gray arrows, including aurone, chalcone, coumarin, dihydrochalcone, dihydroflavonol, and flavan-3,4-diol. Isoflavones are characterized by having the B-ring attached at C_3_ rather than the C_2_ position, and coumarins follow the same structure of isoflavones, except for the double-bond oxygen that is located in C_3_, not in C_4_. Based on Hahlbrock (5) and Crozier et al. (3).

Flavonoids are polyphenolic compounds comprising fifteen carbons, with two aromatic rings (AC and B) connected by a three-carbon bridge, called flavan (Figure 1). About 5,000 flavonoids have been isolated (6), and the important ones are assigned to 12 subclasses, including anthocyanidins, aurone, chalcone, coumarin, dihydrochalcone, dihydroflavonol, flavan-3,4-diol, flavan-3-ol, flavanones, flavones, flavonols, and isoflavones (5, 7). Polyphenolics (e.g., tannins) comprise a significant subclass of flavonoids (Figure 1). Condensed tannins have been extensively used in ruminants because of their ability to reduce methane (CH_4_) emissions (8, 9); shift protein digestion from the rumen to the small intestine (10, 11); improve the maternal environment and reproductive efficiency (i.e., ovulation, scanning, pregnancy, and fecundity rates) (12); support early embryonic survival (13); enhance embryo and fetal development, lambing rates, and lamb survival from birth to weaning (12); and trigger blood cell counts and the immune system response (14), among many other applications (1, 15, 16). Given the broad and sometimes incomplete understanding of CT's impact on the rumen's fermentation dynamics, interest has intensified in their ability to alter animal products' nutritional and organoleptic characteristics. The modulation of ruminal biohydrogenation with consequent alteration of the fatty acid composition of milk and meat is perceived as beneficial to humans because of the relative increase of omega-3, *trans*-11, and conjugated linoleic and linolenic fatty acids (17). Similarly, of particular interest is the ability of CT to reduce gastrointestinal parasite burdens (14, 18–20) given growing concerns of pharmaceutical antiparasitic resistance in grazing ruminants (21) due to their continuous, and sometimes unnecessary, treatment with ivermectin, a macrocyclic lactone.

Approximately 30,000 terpenoids compounds have been identified (6). Among these, saponins are classified into triterpene or steroidal glycosides, having many different bio-physicochemical properties. Most studies in ruminants have focused only on saponins' nutritional aspects to prevent digestive disorders when used as feed additives (22). Plant extract is the typical form adopted to achieve the desired phytochemical compound, and five saponin-rich plants have been consistently examined: *Camellia sinensis* L. (23, 24), *Quillaja saponaria* (25, 26), and *Sapindus rarak* DC.—lerak (27, 28) and *Sapindus saponaria* L.—soapberry (29, 30) with a triterpenoid structure; and *Yucca schidigera* (31, 32) with a steroidal nucleus.

In the last 15 years, some advancements in phytochemical research have been motivated by governmental regulations focusing on public health interests, especially those related to antimicrobial resistance (AMR) due to the broad and unbridled use of antibiotics in animal production as well as poorly controlled use for treating humans (16). Therefore, effective antibiotics replacements, including EO (33), such as allicin (garlic extract), carvacrol (oregano extract), cinnamaldehyde (cinnamon extract), and thymol (thyme extract), have been extensively investigated in broilers, pigs, and aquaculture (34, 35) due to their bacteriostatic and bactericidal properties. However, ruminant studies, including volatile terpenoids as EO constituents, have yielded mixed results (34, 36).

Alkaloids are nitrogen-based chemical compounds synthesized by plants for defensive purposes against predation by an offending organism, such as microorganisms, insects, herbivores, and sometimes, even other plants. Besides the deterrence of predation, there is growing evidence that alkaloids are also produced to harm the offending organism's growth and development through allelopathic action (37). Their toxic effect depends on their type and the amount consumed by the animal, but its primary purpose is to repel feeding via visual or olfactory signals (38). After ingestion and absorption, alkaloids can cause physiological and metabolic changes in the offending organism. Alkaloids can also be produced by animals, insects, and marine vertebrates, although plant extracts are the primary source that has been extensively studied (39). Most studies with alkaloids are related to their toxicological effect on animals rather than their phytochemical feed additive properties. Research on alkaloid pharmaceutical properties in humans was initiated in the 1980s (40). By the early 1990s, about 10,000 alkaloids were cataloged (41), including aconitine (anti-rheumatism), atropine (antispasmodic), caffeine (a stimulant), codeine (analgesic), ephedrine (decongestant), ergotamine (migraine), hydrastine (lower gastrointestinal disorders), and morphine (pain killer) to list a few (42). To date, more than 20,000 alkaloids have been isolated (6). However, few studies were conducted with domesticated animals to improve their production performance, or alkaloids have acted as ghost phytochemicals with unknown biological importance.

This review aims to discuss important nutritional and methodological aspects of major phytochemicals relevant to ruminant production, including flavonoids (e.g., polyphenolics), which comprise 9% of phytochemicals (5,000 in 55,000), terpenoids (e.g., saponins, EO, and fat-soluble vitamins), which contain 55% of phytochemicals (30,000 in 55,000), and alkaloids, which comprise 36% of phytochemicals (20,000 in 55,000).

## Polyphenolics

### Classification and Definitions

Polyphenolic plant secondary metabolites are ubiquitous throughout the plant kingdom. Tannins are a subclass in terrestrial plants broadly categorized into two major compounds: CT and HT. Hydrolyzable tannins are esters of gallic acid with a polyol core molecule, commonly glucose, and might be further categorized into ellagitannin, gallotannin, and galloglucose subclasses. Condensed tannins are polymers of flavan-3-ol (Figure 1) with subunits categorized as catechin, epicatechin, gallocatechin, or epigallocatechin. The diversity of the chemical structures of CT is vast. When considering the multiple subunit and bond types, a simple trimer could represent 1 of nearly 600 different isomers (43). Both CT and HT bind and precipitate protein via hydrogen bonding and hydrophobic interactions (44), a defining characteristic in tannin-ruminant animal interactions.

### Nutritional Importance

The chemical properties of tannins contribute to nutritional and antinutritional effects on ruminant animals. The nutritional importance of tannins largely depends on their ability to bind to macromolecules and mineral nutrients. Condensed tannins and HT readily bind to dietary proteins in ruminants (45, 46) and interact with dietary lipids (47), polysaccharides (48), and metal ions (49). Tannins also alter microbiomes and inhibit microbial and enzymatic activity in the rumen (50, 51) and during the ensiling process (52).

Hydrolyzable tannins have often been regarded as potentially toxic, antinutritional plant secondary metabolites due to their tendency to be degraded in the rumen and absorbed by ruminants (53). More recently, however, potential benefits of HT on ruminant animal production systems have been reported (54–56). Unlike HT, CT have generally been considered as non-degradable by rumen microbes (57). However, possible ruminal degradation and total tract disappearance of CT have been reported. Robbins et al. (58) reported only 75% of CT consumed was recovered in feces of domestic sheep compared to >90% recovery in mule deer. More recently, and using much more sophisticated measuring systems, Kronberg et al. (59) determined that more than 90% of the CT consumed by sheep were degraded. Conversely, Desrues et al. (60) recovered all CT from sainfoin following total tract passage through cattle. Like many biological activities driven by CT, degradation in the ruminant digestive tract is likely dependent upon plant species, tannin type, and chemical structure. The variation in the survival of CT through the digestive tract point to the need for the strategical application of CT (i.e., nutritional vs. antiparasitic effects, rumen vs. post-rumen activity, CH_4_ abatement vs. rumen protected N) rather than the commonly used “shotgun” approach. The fate of CT in ruminants and associated nutritional implications should continue to be a future focus of research on physiological and modeling research (15).

#### Ruminal Fermentation

Polyphenolic phytochemicals potentially offer numerous benefits to ruminant animal production. The most notable of those benefits is rumen microbiome modifiers (61) to alter gaseous emissions (62) and improve animal production (63). Much of the recent research on tannins has focused on the topic of rumen modification to mitigate greenhouse gas emissions and improve the N-use efficiency of ruminant livestock. The majority of this research has focused on the application of CT, but increased research interest in HT is becoming more evident.

Our knowledge and potential application of tannins in production scenarios are hindered due to a lack of understanding of how tannins interact with substrate and microbes in the rumen. Currently, CT are believed to reduce CH_4_ production in the rumen by combining three possible mechanisms (43): (1) the formation of CT complexes with fermentable macromolecules and microbial enzymes, reducing the availability of substrates to microbial degradation, (2) the direct interaction between microbes and CT, resultant of CT binding to microbial lipopolysaccharide, and (3) CT subunits degrade in the rumen and become hydrogen sinks, reducing the hydrogen available to form CH_4_ gas. The hydrogen-sink hypothesis has been demonstrated with catechin monomer subunits *in vitro* by Becker et al. (64). However, tannin scientists have yet to reveal the possibility that CT polymers undergo the necessary degradation in the rumen to become hydrogen sinks. Similar to CT, HT is thought to reduce enteric CH_4_ by directly interacting with microbes or acting as a hydrogen sink (65). However, it is not believed that HT reduce CH_4_ by decreasing substrate availability as a concomitant decrease in CH_4_, and fiber digestion is typically not observed (66). However, our assumptions of how tannins behave in the rumen are continually evolving and require technological advancements and modeling techniques to understand the dynamic relationship better.

Recent research has focused on the application of respirometry methodologies to increase our understanding of the effects tannins have on CH_4_ emissions *in vivo*. However, *in vivo* research has been inconsistent, with discrepancies among CT and HT studies being indicative of complex associations. For example, the use of quebracho CT extract has resulted in reports of reduced CH_4_ emissions (62, 67) and no effect (68). Of these studies, beneficial effects were observed in those that fed a roughage diet and higher rates of CT (>1.5% DM). This may be, at least in part, an effect of CT rate or diet type but is likely a combination of the two; however, we lack conclusive data to understand this complex relationship. Similarly, HT in ruminant diets has also demonstrated varied results for CH_4_ emissions. Recent work showed that gallic acid, an HT derivative, and tannic acid reduced CH_4_ emissions in beef cattle (54, 69), whereas HT from chestnut appears to have little or no effect (70). However, once again, we lack adequate data to conclude the reason for these differences. The discrepancies among CT and HT studies appear to point to a dynamic relationship among a variety of factors, including the chemical structure of the tannin, tannin inclusion rate, base diet (i.e., forage vs. concentrate), and animal species and stage of production (i.e., maintenance, growth, fattening, or lactation).

To garner a better understanding of the dynamic relationship among factors affecting tannin efficacy, *in vitro* gas production techniques have been used to screen tannin-rich forages for their potential to alter fermentation patterns and reduce CH_4_ emissions by ruminants. This approach has proven to be a cost-effective and time-saving tool (71, 72). These techniques are especially useful when exploring domestication of wild types of perennial prairie legumes (73), investigating increased utilization of arboreal plant resources as forage (74), or when seeking the value of feeding invasive plant species to ruminants (75, 76). However, there is often some disparity between *in vitro* and *in vivo* CH_4_ production (77). To better understand the real impact of tannins on ruminant nutrition, long-term and conclusive *in vivo* or *in situ* studies must be conducted to calibrate *in vitro* data. We must enable the application of *in vitro* methods to provide a rapid determination of tannin feasibility in various production systems.

#### Post-rumen Digestion

Ruminant animals are generally considered inefficient at converting ingested protein into an animal product due to a large portion being lost as NH_3_ in the rumen. The efficiency of N use and retention by ruminants can be improved by either slowing the degradation rate of protein to enhance synchrony with carbohydrates or increasing rumen undegradable protein in the diet (78). Much of the interest in tannins revolve around the prospect of possible degradation and absorption of rumen undegradable protein following dissociation from tannin-protein complexes post-rumen. Condensed tannins readily decrease ruminal N digestibility (79), resulting in reduced urinary N excretion (62, 80) with a concomitant increase in fecal-N excretion and a possible reduction in excreta gas emissions (81). This shift in the site of N excretion might represent a decrease in N retention. However, some have reported increases in the efficiency of protein utilization expressed as weight gain per protein intake due to CT inclusion in the diet (82). Hydrolyzable tannins also bind and precipitate proteins, possibly increasing post-rumen availability of N, but they also offer the potential to slow the ruminal degradation of N and possibly promote the synchrony of N and carbohydrate degradation. Much like what is observed when feeding CT to ruminants, a shift in N excretion from urine to feces is observed when including HT in the diet (56). While increases in N utilization associated with feeding HT have not been reported, supplementation with gallic acid may decrease urinary N excretion without negatively impacting N digestibility (54).

The ability to shift the route of N excretion from the urine to the feces without sacrificing N digestibility is increasing in interest due to excreta's contribution to total livestock emissions. The feeding of CT has demonstrated the potential to decrease fecal gas emissions (81, 83) and reduce fecal urease activity (84). Similarly, nitrous oxide emissions from urine were reduced when gallic acid was fed (51). Once again, we lack adequate data to assume the mechanism(s) that alter emissions or that the observed alterations in excretion route and excreta emissions will improve overall emission status. However, based upon the positive results observed in the limited number of studies performed, research into the effect of tannins on excreta emissions warrants greater focus.

### Gastrointestinal Nematodes

Gastrointestinal nematode (GIN) and other gastrointestinal parasite infections negatively impact ruminant nutrition. Both small and large ruminants are affected, but internal parasites are especially detrimental to small ruminants, including sheep and goats. Legume CT, particularly those from sericea lespedeza (*Lespedeza juncea* var. sericea), demonstrate anthelmintic activity against GIN parasites in small ruminants (85). Sericea lespedeza (*Lespedeza cuneata*) (86) and quebracho (*Schinopsis* sp.) (87) CT also inhibits *Eimeria* spp. in goats, which are responsible for coccidiosis. Condensed tannins may also be efficacious as an anthelmintic against common cattle parasites *Cooperia oncophora* and *Ostertagia* spp. (88).

Increased use of the larval exsheathment assay has led to the screening of novel forage CT for anthelmintic activity (89). *In vitro* screening for the potential anthelmintic activity of tannin-rich forages is not limited to CT. Concentrations of 25 mg HT/ml effectively kill *Haemonchus contortus in vitro* (55). Gallic acid reportedly demonstrates egg hatch inhibition against GIN that commonly infect cattle (90).

An important question that cannot be answered using *in vitro* techniques is what are the negative nutritional and toxic implications, if any, of feeding HT to ruminants for GIN control? Therefore, *in vivo* research must follow reports of positive impacts of CT but especially HT to confirm anthelmintic activity without detriment due to phytochemical toxicity. Some hypothesize that parasitized ruminants will intentionally select forages with anthelmintic properties (i.e., tannins). Some evidence of ruminant self-medication by selecting for tannin-rich forages when parasitized by GIN has been reported (91). More often than not, however, the self-medication hypothesis is not confirmed (92, 93).

#### Why and When Do Tannins Work?

The question of when and why tannins positively impact ruminant nutrition is a difficult one to answer. Tannin bioactivity, especially that of CT, is often plant-specific. The mechanisms of action for tannin biological activities, such as ruminal CH_4_ mitigation, reducing rumen proteolysis, or inhibiting GIN, are mostly unknown. The mechanism for one biological activity likely differs from that of another.

There is evidence that structurally recalcitrant tannins are most effective in modifying fermentation and reduce CH_4_. For example, CT from *Acacia angustissima* var. *hirta* are highly effective at reducing enteric CH_4_ production (73). The undegradable 5-deoxy flavan-3-ol structure of the *Acacia* CT likely contributes to its ability to mitigate CH_4_ formation during fermentation (94). Additionally, tannins' antioxidant activity is positively correlated (*r* > 0.90) to ruminal CH_4_ emission (94), suggesting that antioxidant activity at least contributes to the mechanism of action involved in CT-CH_4_ mitigation.

The ability of tannins to bind and precipitate protein, and potentially create rumen undegradable protein, may depend on factors associated with chemical structure and conformation, the pH in which the tannin-protein interaction occurs, and the herbivore's ability to bind tannins with salivary proteins during mastication and rumination. The structural diversity of tannins adds to the difficulty of determining the impact of specific structural characteristics on tannin-protein interactions. Structural attributes of CT, including the mean degree of polymerization, stereochemistry, and prodelphinidin-to-procyanidin ratio, sometimes do not explain protein-tannin interactions (95). However, some reports suggest that large prodelphinidin-based CT demonstrate greater protein precipitating capacity than large procyanidin-based CT (96). Recently, the impact of the increased mean degree of polymerization and inter-flavan bond type on protein precipitation capacity has been confirmed (97). Similar to factors affecting protein precipitation by CT, larger polymers of HT demonstrate greater protein precipitating capacity than monomeric forms (98).

The pH is an important factor affecting the protein precipitating capacity of tannins; the closer to the isoelectric point of the protein, the greater the protein precipitation capacity (99, 100). Much of what we know about the role of pH in CT-protein interactions supports the hypothesis that protein-tannin complexes dissociate in acidic environments (such as in the abomasum of ruminants), leading to protein degradation and subsequent amino acid absorption in the small intestine. As the pH of the environment where tannin-protein complexes occur becomes more acidic relative to the isoelectric point of the protein, the protein precipitating capacity of tannins decreases (100). Accordingly, when the environment is less than pH 5, tannin-protein complexation may be minimal (101).

The neutralizing effect of proline-rich protein in saliva has long-been hypothesized (102). Many browsing herbivores that readily consume tannins do not produce saliva that contains proline (103). Despite a lack of proline, some browsing ruminants (i.e., goats) can bind tannins with salivary proteins (104), suggesting that proline is not a requisite for all salivary protein-tannin interactions.

An explanation for when and why tannins are useful anthelmintics continues to be elusive. Much of the literature suggests the efficacy of both CT and HT against GIN is dose-dependent (55, 89), such that greater concentrations of tannin result in more significant anthelmintic effects. Tannin concentrations vary within species based on plant maturity, which is another factor to consider. In some species, mature plants produce lower tannin concentrations than immature plants (76, 105), whereas others may increase or remain unchanged with maturity (105). However, it is crucial to fully understand the CT concentration at different seasonal growth stages in a given plant species (106) to maximize their ontogenic phytochemical characteristics on sustainable ruminant production systems (8).

#### Why Do Tannins Not Work?

Dietary tannins do not always affect the nutritional status of the ruminant animal. There are many possible reasons for this. If the forage or feed resource is too low in tannin concentration, little, if any nutritional impact will be observed. The tannin's chemical structure produced by a given plant can determine whether or not the phytochemical is effective at eliciting a desired animal response. Modes of action of tannins also differ for different activities such that the type and structure of tannin used to elicit one nutritional response may not be useful for that of another. Another challenge occurs when feeding highly bioactive tannin-rich forages. The animal may reject tannin-rich forage due to reduced palatability due to salivary protein binding and astringency.

### Future Perspectives

There is still much to learn about how CT and HT affect ruminant animal nutrition. Much of what we understand about tannin impacts on ruminant nutrition is the result of *in vitro* studies. While *in vitro* assays are excellent screening tools, more *in vivo* confirmation of research findings is needed to move tannin science from use-inspired basic research to application. A significant challenge to this progress is the lack of domesticated (cultivated) plants rich in bioactive tannins. As a result, the availability of plant material suited for many ruminant producing regions is limited. Even when the seed is commercially available, it is often cost-prohibitive due to the limited supply and labor required to collect undomesticated species.

Future research should emphasize the strategical application of tannins rather than the current “shotgun” approach from a nutritional perspective. Much of the previous and recent research has emphasized directly inhibiting enteric CH_4_ production and increasing rumen undegradable protein. However, there is potential to utilize some tannins' degradation to reduce CH_4_ via hydrogen-sink and increase N-use efficiency by improving nutrient synchrony. There are opportunities to exploit tannins' antioxidant properties, particularly immunomodulatory effects, thermal stress, and human-health products. Tannins' influence on excreta emissions requires attention, but ultimately we need to understand better how excreta from animals consuming tannins alters soil fertility, soil microbiota, and plant growth.

Despite deficiencies in current knowledge about nutritional implications in ruminant animals, polyphenolic phytochemicals (i.e., tannins) have great potential as a tool in ruminant production systems. Further investment in plant breeding and domestication efforts, as well as research efforts to further elucidate how tannins impact ruminant nutrition and system processes, will be necessary to realize the full potential of these important phytochemicals.

## Terpenes

### Biosynthesis and Functionality

The bitter-taste, emulsifying, foaming, non-ionic, non-volatile, membranolytic, surfactant, and structurally diverse saponins (glycosides) are low molecular weight (1,000–1,500 Da) secondary natural compounds in food and non-food plants (107–109), marine plants (110) and animal lineages, including invertebrate sea cucumber species (111, 112). Chemically, glycoside saponin biosynthesis begins with the catalyzation of acetyl co-enzyme A to isopentenyl pyrophosphate units generated by the multistep mevalonate 3-hydroxy-3-methylglutaryl-CoA reductase (113), a common route to the synthesis of cholesterol and some steroids (114).

Saponins comprise the hydrophobic aglycone (sapogenin) structure that is linked to polar functional groups and attached via a 3-C chain structure to an individual or multiple hydrophilic sugars (i.e., arabinose, galactose, glucose, glucuronic acid, methylpentose, rhamnose, or xylose) (115, 116) and moieties (i.e., glycones) (117, 118). Aglycones are subject to gene encode enzyme-mediated (i.e., cytochrome P450-dependent glycosyltransferases, monooxygenases, and others) (119) change (i.e., acylation, hydroxylation, glycosylation, oxidation, and substitution) (119, 120) to form a varied group of compounds (121).

Saponins are chemically categorized into two groups: triterpene or steroidal. Following the isoprenoid pathway, the aglycone splits into pentacyclic triterpenoid saponins (**TPS**) with a 30-C aglycone core by cyclization of 2,3-oxidosqualene (113, 117, 122), yielding the first group of saponins. The second group is related to the biosynthetic pathway of tetracyclic steroidal metabolites to a 27-C aglycone backbone (114, 117, 120) with a 5-ring furostane or a 6-ring spirostane skeleton (123) involving oxygenations and glycosylations (117).

Although in the presence of other phytochemistry (124), saponin mixture in a single plant species occurs (120, 121, 125), such as cucurbitane, cycloartane, dammarane, holostane, hopane, lanostane, lupane, oleanane, tirucallane, taraxastane, tirucallane, and ursane TPS types (107, 126) have been identified in more than 500 plant species (114). Within a hundred family-group plants, the Anacardiaceae, Araliaceae, Combretaceae, Compositae Campunalaceae, Caryophyllaceae, Leguminosae, Polygalacea, Sapindaceae, Theaceae, and Verbenaceae families, their genera and species attract more attention (114, 127–131).

In angiosperm monocotyledons and angiosperm dicotyledons plants, the variation, composition, concentration, distribution, and differential bio-activity of TPS are influenced by plant growth, agronomic and genotype-environmental interactions (132–134). Moreover, TPS-plant storage, physical milling, TPS separation, and the bio-accessibility of metabolites in the form of concentrated extracts, derivatives, or food additives to facilitate human-animal utilization may modify aglycones' structure and their bio-physiological, nutraceutical, and pharmaceutical activities (121, 124, 135).

Although paths for those roles are not well-understood and despite differences in chemical structures, different activities exist for TPS, including adjuvant (136), antibacterial (137, 138), antidiabetic (139), antifungal (140–142), anti-inflammatory (123, 125), antioxidative (109, 143), antiprotozoal (144–146), antiproliferative (147), antiviral (148, 149), cardiotonic and cardioprotective (122), and cytotoxic (127, 128, 150) effects have been reported. Additionally, TPS have also exhibited other functional properties, such as food-additive in flavorings (26), gastroprotective (151, 152), hemolytic (153), hepatic (139, 149), immunologic (123, 154), insecticide (155, 156), anti-obesity therapeutic potential (111, 116, 157, 158), neuroprotective (159), vermicide (160), and emulsifier and stabilizer of the nanosuspensions (161, 162).

### Nutritional Importance

Central to TPS's bio-physicochemical network of interactions, the nutritional significance of TPS for ruminants stems largely from their digestive and methanogenic significance (163). Consequently, using *Medicago sativa* L. (alfalfa) and *C. sinensis* L. (tea plant) as examples, this review will be limited to considering certain aspects of the bio-metabolic and rumen microbial shifts in sheep and cattle derived from TPS supplementation, which are not entirely consistent and understood. Compared to non-supplemented diets, Table 1 has a comparative overview of digestive function reaction to alfalfa-TPS (26.9–601.3 mg/g extract) intraruminal or feed-mixed supplemented [10.6–800 mg TPS/kg body weight (**BW**)] in different breeds and BW (42–60 kg) of sheep between 14 and 90 days.

**Table 1:** Effects of triterpenoid saponin (TPS) supplementation on several ruminal and total gastrointestinal tract parameters^1^.

**Plant species^**2**^**	**TPS-animal interaction**					**Digestive parameters**							
	**Extract**	**TPSC**	**Animal**	**mg/kg BW**	**TPS:DMI**	**RM**	**RPC**	**PMO**	**OMD**	**NDFD**	**TVFA**	**CPP**	**DMP**
*Medicago sativa* L.^a^	Root	27.8	Sheep^‡^^⊺^	200	1	1.74	+22	0	−1	−2	+6	−42	+6
				400	2	1.67	+21	+4	−7	−8	+3	−81	+5
				800	3	1.67	+53	+25	−12	−12	+17	−88	−2
					**TPS:DM**	**RTR**	**TMRT**	**DMD**	**OMD**	**HEMD**	**CELD**	**TVFA**	**CPP**
*M. sativa*^b^	Plant	26.9	Sheep^†^	10.6^*^	2	−6	0	−1	−1	−2	−6	+1	−37
				21.4^⁑^	4	−3	−2	0	0	−1	+4	−6	−47
				10.6	2	−24	+10	+6	+1	+35	+32	−19	−33
				21.4	4	−23	+7	+9	+2	+28	+40	−28	−55
					**TPS:DMI**			**DMD**	**NDFD**	**ADFD**	**EED**	**CPD**	
*M. sativa*^c^	Leaf-root	601.3	Sheep	12.0	0.04			−1	−15	+15	0	+2	
				24.0	0.08			+11	+22	+17	+2	+9	
				47.1	0.16			+4	+1	−24	−2	+3	
				94.3	0.32			−3	−8	−15	−5	−4	

1 *Ratios of TPS to dry matter (DM) (TPS:DM) or TPS to DM intake (DMI) (TPS:DMI); rumen motility (RM, n/min)^†^; rumen pressure change (RPC, mm Hg)^†^; ruminal turnover rate (RTR, %/h); total mean retention time (TMRT, h); particulate matter outflow (PMO, g/d); total-tract crude protein (CP), DM, ether extract (EE), organic matter (OM), neutral detergent fiber (NDF), acid detergent fiber (ADF), hemicellulose (HEC), and cellulose (CEL) digestibilities (g/100 g); total volatile fatty acids (TVFA, mmol/L); ciliate protozoal populations(^‡^CPP × 10^5^/ml); and daily methane production (DMP). The notations + refers to an increase and – refers to a decrease in percentage values relative to non-TPS supplemented diets data in each experiment. Triterpenoid saponin concentration (TPSC) is presented in mg/g of M. sativa plant extract; and mg/kg DM of M. sativa root extract, and M. sativa leaf-root commercial extract product*.

2 *a = Klita et al. (115) in which intraruminal TPS extract supplementation was conducted for 14 days*.

† *Measured on day 11*.

⊺ Methane measurements (24 h) on day 12 based on indirect calorimetry and respiratory hoods from 4 Suffolk wethers. b = Lu and Jorgensen (164) in which ^*^ roughage and ^⁑^concentrate diets fed to wethers subject to intraruminal daily supplementation of TPS during 14 days. c = Liu et al. (108) in which TPS-supplemented concentrate plus roughage diet were fed twice daily to 10 Hu male-lamb groups (n = 5) during 90 days.

Based on the use of 17.8–35.9 mg TPS/g extract, an intraruminal increasing TPS-dose in wethers fed roughage diets resulted in a less disturbed digestive system than the digestive responses of intraruminal supplemented wethers fed concentrate diets (164). However, using 27.8 mg TPS/g extract, compared to the lowest intraruminal dose of 200 mg/kg BW in Suffolk wethers fed grass-hay, 800 mg/kg BW administered intraruminally increased rumen pressure, particulate matter outflow, and VFA concentration by 25, 25, and 10%, respectively (115). This effect was further associated with a reduction in organic matter (11%) and neutral detergent fiber (10%) total tract digestibilities, ciliate protozoa populations (**CPP**; 80%), and daily CH_4_ production (8%) (115).

There is limited experimental data on the use of TPS on animal production under mid to long-term management. However, Liu et al. (108) demonstrated complementary opportunities for both physio-metabolism and production evaluation. These authors indicated that a high-TPS concentration extract shifted from 0.04 to 0.08 TPS-to-dry matter intake (**DMI**) ratio in concentrate plus roughage diets used by Hu male-lambs during 90 days, yielded a 12, 44, 2, 2, and 7% increase in dry matter (**DM**), neutral and acid detergent fibers, ether extract, and crude protein digestibilities, respectively. Nevertheless, when the TPS supplementation increased from 24 to 94.3 mg/kg BW, the effects on DM, neutral and acid detergent fibers, ether extract, and crude protein digestibility decreased by 12, 44, 2, 2, and 7%, respectively. These effects were also associated with an 8% reduction in daily BW gains.

These studies illustrate how sheep responses can be influenced by motivated, focused action. However, the long-range vision to shape or reshape TPS's use and ensure its relevance to small ruminant needs a particular combination of knowledge and perspectives. It should equate the sheep feed industry interest with clinical science in the context of a deepening sense of animal practice responsibilities to concomitantly address societal needs and ecosystem environmental challenges.

Overall, we can only speculate that the TPS-extract source within the same plant species, the extraction method, compound composition, concentration and dose, way and time of supplementation, diet type, and sheep genetics refer to the range of variation in the summarized alfalfa-TPS supplementation response in Table 1. Unless such information is forthcoming, there is a risk of limiting factors to benefit from the TPS functional activities described above with sheep if they are susceptible to specific doses in farming grazing conditions.

Table 2 illustrates how cattle and sheep respond to TPS supplementation. It illustrates the impact of TPS doses from tea seeds and alfalfa extract sources on fermentative, microbial, and blood parameters of Brahman (*Bos indicus*) and crossbred *B. indicus* cattle (234–364 kg) and sheep (41.7–42.5 kg). The approach is justifiable because, in the current and post-COVID challenges, it is unlikely that individual research could undertake simultaneous cattle-sheep TPS supplementation assessments. However, it would be possible for cooperative research across the livestock industry to justify the expense involving additional knowledge gains.

**Table 2:** The effects of supplementing triterpenoid saponin (TPS) from *Camellia sinensis* L. or *Medicago sativa* L. on digestive and blood profiles of ^†^Belmont Red Composite [Africander (African Sanga) × Brahman (*Bos indicus*) × Hereford-Shorthorn (3/4 *B. taurus*)] and ^⸸^Brahman steers, and ^‡^Dorper crossbred × thin-tailed Han ewes, ^ϯ^Hu rams, ^ͳ^Huzhou lambs and ^*^Hu male-lambs^a^.

**Parameters^**b**^, ^**c**^**	***C. sinensis***^**b**^, ^**c**^											
	**Cattle**^**†**^					**Sheep**^**‡**^		**Sheep**^**ϯ**^			**Sheep**^**ͳ**^	
**TPS mg/kg BW**	**31.5**	**44.3**	Post-TPS			28.6		83.7			112	
**TPS:DMI**	**0.14**	**0.22**	Post-TPS			0.13		0.18			0.30	
**TVFA**	**+3**	**−2**	−2			+16		−3			+13	
**CPP**	**+99**	**+190**	−5			−16		−42			−41	
**DMP**	**+6**	**+3**	−16			+2		−11			−27	
**Animal species**	***C. sinensis*** **serum biochemistry**											
	**TPS mg/kg BW**	**TPS:DMI**	**CL**	**K**	**Na:K**	**I**	**GLU**	**CHO**	**UM**	**GGT**	**ALP**	**AST**
Cattle^⸸^	22.7	0.11	−1	−12	+12	−23	−15	+5	−29	+5	+5	−5
	44.2	0.21	+1	−11	+12	−21	−15	0	−17	+10	+10	−7
	64.9	0.30	+3	−12	+13	−22	+3	+5	−20	+17	+17	0
	***M. sativa*** **plasma profile**											
			**GH**	**IGF-1**	**T3**	**T4**	**GLU**	**CHO**	**UN**	**TRG**	**ALT**	**AST**
Sheep^*^	12.0	0.04	+38	+32	+77	+42	+20	+2	+5	−2	+46	+31
	24.0	0.08	−9	−5	+22	−9	+16	−7	−30	−35	+49	+27
	47.1	0.16	+8	+2	+11	−18	+20	−9	−44	−68	+81	−27
	94.3	0.32	+5	−8	+40	+4	+21	−3	−35	+3	+98	−28

a *Positive and negative percentage data refer to non-TPS supplemented diets in each experiment*.

b *Ciliate protozoal populations (^†^CPP Log × 10^8^/ml rumen fluid,^ͳ^ CPP a % of total bacterial 16 S rDNA, ^ϯ^ CCP × 10^5^/ml,^‡^CCP × 10^7^/ml), daily methane (CH_4_) production (DMP), dry matter intake (DMI), body weight (BW), total volatile fatty acids (TVFA, mmol/L). Serum electrolytes and minerals [mmol/L; chloride (CL), potassium (K), sodium to potassium ratio (Na:K), iron (I, μml/L)]. Metabolites [mml/L; cholesterol (CHO), glucose (GLU)]. Renal function [mmol/L; urea nitrogen (UN)]. Enzimes [IU/L; alkaline phosphatase (ALP), aspartate aminotransferase (AST), γ-glutamyl transferase (GGT)]. Plasma hormones [ng/mL; growth hormone (GH), insulin-like growth factor-1 (IGF-1); mmol/L; tri-iodothyronine (T3), and thyroxine (T4)]. Metabolites [mg/dL; urea nitrogen (UN), glucose (GLU); mmol/L; triglyceride (TRG), alanine transaminase (ALT), aspartate aminotransferase (AST)]*.

c † *= Ramírez-Restrepo et al. (165) in which eight rumen-cannulated steers were progressively supplemented with dissolved tea seed saponin (TSS; 580 mg TPS per g of TSS) mixed in the morning diet during 3 and 4 days. Post-TPS values were recorded 13 days after TPS withdrawal. Individual CH_4_ emissions were measured (48 h) in open-circuit respiratory chambers, recording levels of supplementation of 27.0 and 43.5 mg TPS/kg BW, which are equivalent to ratios of 0.13 and 0.23 TTS:DMI, respectively*.

⸸ *= Ramírez-Restrepo et al. (166) in which after 13.8 mg TPS/kg BW (0.08 TPS:DMI) supplementation during 6 initial days, a gradual increase of intraruminal (four cannulated steers) dissolved TSS supplementation before the morning feeding and mixed in the morning feed (2 non-cannulated steers) was performed during 7, 14, and 16 days, respectively*.

ͳ *= Mao et al. (167) in which 32 lambs fed in two equal parts daily. Open-circuit respiratory chamber measurements (48 h) and microbial populations from four lambs after 60 days trial*.

ϯ *= Zhou et al. (168) in which 12 rumen-fistulated Hu rams fed once a day. Three rumen-fistulated and re-faunated Hu rams supplemented with 1.8 g of TPS for 3 weeks in the basal diet. Open-circuit respiratory chamber measurements (24 h)*.

* *= Liu et al. (108) in which TPS-supplemented concentrate and roughage diets fed AM and PM to 10 Hu male-lamb groups (n = 5) during 3 months; physiological values in the 60–90-day period*.

‡ *= Liu et al. (108) in which 18 primiparous and six rumen-cannulated Dorper × thin-tailed Han crossbred ewes were used and fed supplemented for nutrient digestibility and CH_4_ emissions in open-circuit respiratory systems (Experiment 1, 29 days), and fermentation and microbial ecology examination (Experiment 2, 42 days), respectively*.

As with beef cattle, sheep can cope with increasing doses of tea seed-TPS. A difference is the range of TPS doses tested between large and small ruminants. Another critical difference is the greater emphasis on cattle measurements after TPS withdrawal than on sheep. This has resulted in the interaction among supplementation digestive and fermentative parameters. The summarized data indicate that Ramos-Morales et al. (169) pointed out that TPS does not always reduce CPP. However, this information may not be surprising because saponin functional diversity and biological pathways do not always positively correlate (170). Early on, Dourmashkin et al. (171) provided evidence that saponins at 0.05% concentration modify eukaryotic cell membrane permeability by producing a pore-forming characteristic expected to inhibit both CPP (115) and CH_4_ emissions (172).

Published trials using tea seed-TPS indicated that their anti-methanogenic effect *in vitro* (173) in small ruminants (167, 168, 174) is considered to be a selective saponin-sterol association (175, 176) on protozoa surface (170). Nevertheless, CPP may increase when plant-TPS (145, 177) and low cell-wall carbohydrate diets are fed (178).

Dourmashkin et al. (171) found that saponin-treated cell membrane growth is associated with concentrations above 0.09%. Sidhu and Oakenfull (179) also demonstrated that, when orally fed, saponins are not absorbed into the bloodstream but might modulate mitosis (180, 181) by molecule transport, cell membrane fluidity, and cell proliferation *in vitro* (182) and *in vivo* (183).

Contrary to the transient antiprotozoal effect of TPS (184), a linear increase of protozoal numbers is triggered by increasing tea seed-TPS doses in crossbred Brahman cattle, while a defaunation effect was observed at 13 days post-TPS treatment as shown in Table 2 (165). There, TPS modified the structure of the methanogen community at the subgenus by increasing the numbers of methanogens and decreasing their abundance in the RO and SGMT clades, respectively (185). In parallel, TPS supplementation reduced numbers of protozoal genus *Entodinium* spp. and increased *Euplodinium* and *Polyplastron* genera. The withdraw of TPS supplementation was associated with lower proportions of *Isotricha* and the greater presence of *Metadinium* and *Eudiplodinium* genus (185).

This suggests that, in tropical cattle, TPS may have a high selectivity index for protozoa, without an adaptation of those ciliates and other microbial communities to short-term feeding of TPS. Moreover, it is essential to note that tea seed-TPS as a feed additive appears to exert a differential protozoal and anti-methanogenic effect across Dorper × thin-tailed Han crossbred ewes, Hu rams, and Huzhou lambs (Table 2). With these facts in mind, readers are directed to Hu et al. (173), Guo et al. (172), Mao et al. (167), Zhou et al. (168), Wang et al. (186), and Liu et al. (187) for the detailed complementary impact of TPS on rumen ecology and extend of nutrient digestion. Together, these findings mirror the belief that further research is required to understand better multifaceted TPS supplementation effects associated with the breed, sex, and animal category sound interactions.

### Future Perspectives

Although in our research no comparison of patterns of CH_4_ emissions was performed between a single and two equal daily portions of TPS supplementation, there is little doubt that the circadian rhythm of CH_4_ emissions from steers after the morning non-supplemented and TPS-supplemented diets (165) is consistent with that observed in twice-daily TPS-supplemented sheep (167, 168, 188) and cattle fed Rhodes grass (*Chloris gayana* Kunth) *ad libitum* (189). Conversely, the current review provides evidence that forage diets fed to ruminants could modulate the animal response to TPS-sources inclusion in tropical agriculture (177, 190–192). However, this reason may be further explained by capturing TPS supplementation advantages in seasonal nutrition, fermentability, and methanogenic indices of forages (71, 72). A sustainable ruminant industry should consider three questions. How long does the TPS-protozoal selective effect in the rumen ecosystem of tropical cattle last? Is this physio-metabolic response opening the possibility that tea seed-TPS may reduce cattle CH_4_ emissions in the long-term rather than as an immediate abatement? Should we investigate the effects of very low TPS concentration additives and/or far lower TPS:DM ratios on ruminants to achieve target microbial community profiles without significant associated meta-physiological disturbances?

Few ruminant studies beyond methanogenesis have focused on complementary clinical responses to TPS supplementation (Tables 1, 2) to understand or confirm pharmacological discoveries, phytochemical screening, safety, and efficiency of therapies, and *in vitro* findings. In particular, safety and tolerability studies have demonstrated that Brahman (166) and Belmont Red Composite [Africander (African Sanga) × Brahman × Hereford-Shorthorn (3/4 *B. taurus*)] (165) steers tolerate on average 32.2 ± 16.61 and 27.3 ± 13.53 mg/kg BW of TPS supplementation during 23 and 20 days, respectively. This for each breed is ~6.4 and 4.5 vs. 5.5 and 3.8-fold the non-toxicological effect levels in mice (i.e., subcutaneous injection) (117) and dogs (i.e., intramuscular route) (193), respectively.

However, as low TPS doses in Brahman (13.8 ± 0.64 mg/kg BW) and Belmont Red Composite (9.2 ± 0.35 mg/kg BW) steers are 1.9 and 1.2%; respectively, of the canines long-term daily administration (i.e., 26 weeks), this variation might facilitate further efforts to clarify biological constraints and a vision of improved farming practices. In parallel, TPS effects on animal behavior and health indicated that the administration at 0.42 ± 0.013% of the DMI to Brahman steers remarkable reduced DMI, and developed primary tympany and enteritis.

Although that high dose was not tested on Belmont Red Composite steers, a similar clinical pattern of symptoms but a lower magnitude were experienced when TPS doses achieved between 0.10 and 0.14 ± 0.003% on the DMI. This was consistent with other studies (194, 195) that reported that some TPS might disrupt endothelial permeability, infiltration of cellular systems, and active nutrient transport, and nutrient uptake in the gut. This likely involves a sequential cascade involving cytokines, chemokines, reactive oxygen species expressions, and several intracellular signaling pathways, to name a few (196). However, those cattle dose-dependent effects contrast Klita's et al. (115) reports that sheep have a lethargic feeding behavior and lack of rumination associated with intra-ruminal TPS:DMI ratios of 4 and 8%.

The interaction between TPS and the functional capacity of organs and body systems can produce relatively complicated outcomes. Table 2 underpins blood test differentiation between TPS-plant sources and animal species. That strategy should, in turn, allow greater understanding of significant differences in blood biochemistry and biological drivers between non-cannulated and cannulated cattle after TPS supplementation (166). Based on the evidence provided here, it appears that such physiological associations could be the vehicle to spread knowledge and refine and collect prolonged assessments to ensure practical use of TPS additives.

Collectively, in response to the natural structure of TPS and their related sapogenins (126, 169, 170, 184), possible reasons for the observed differences within bovids are the pharmacodynamic and pharmacokinetic profile expressions of the host physiological system (197, 198). This is likely characterized in healthy animals by differential genetic and metabolic binding, inter-individual variability, cellular and molecular self-regulatory feedback mechanisms, induction and inhibition of pathways, pharmaco-genomics, and pharmaco-metabolomics (199).

However, supported by the heterogeneity of systemic reactions shown in Tables 1, 2, it is suggested that a broad medical approach in future studies is critical to understanding TPS supplementation throughout the interrelationships within and between ruminant species, breeds, and crossbred animals. Medicine will benefit from increased knowledge of more significant or down-regulation expression of signal transducers, transcription factors, membrane proteins, ion channels, and mitochondrial enzymes in cell lines (200, 201). Such observations further indicate the relevance of complementary microbiota analysis to understand the impact on ruminal ecology, methanogenesis, and animal physiological functioning following clinical-relevant TSS-supplementation and at withdrawal endpoints.

In summary, although over the last years, review research advances in TPS have been evident (27, 162, 202–208), the disparities in physicochemical characteristics of close and non-closely intermediate related compounds in TPS-containing plants (209–211) from one to another material depends on the vast structural diversity of TPS molecules (131, 212). Therefore, feasible investigations should focus on TPS physio-metabolic interactions after ingestion to elucidate complex interactions with the diet's nutritive value and substantial variation in gastrointestinal microflora and animal metabolisms. This is reasonably straightforward in intermolecular forces, genetic-molecular animal predispositions, cellular signaling frameworks, intra-cellular-matric chemoreceptors, metabolic fluxes, multi-enzyme cascade, and morphological changes. The approach across the catalog of TPS-plants, their phytochemical compounds, and interactions will promote secondary compound-physiological-based ruminant models (15), human and animal health, regulatory environments, ecosystems management, and eco-efficient ruminant production.

## Vitamins and Antioxidants

### Types of Vitamins

Various vitamins and related minerals, many of which play critical roles as antioxidants important for growth and health, are sometimes deficient in ruminant diets. Ruminant requirements change with species, class, age, weight, health, and growth performance (213), but much of the research into these requirements are outdated and not representative of current production systems. Vitamin and related mineral deficiencies most often affect animals fed in confinement and only rarely occur in those allowed to graze or browse pastures and rangeland containing abundant, diverse plant species except when soils are severely deficient, as is sometimes the case with Se (214). When deficiencies occur, they are often a result of incorrect ration formulations or antagonistic effects (e.g., K and P, S, and Cu). However, they can be corrected by supplementation, feed changes, or allowing animals access to diverse pastures containing dicotyledenous species, such as legumes. Historically, cattle confined feeding operations have supplemented ruminants at or above published requirements as a preventative measure (215).

In grazing or browsing ruminants, most vitamins and minerals necessary in cellular antioxidant activity can be ingested from fresh plant material. In turn, these are transferred to ruminant products; dairy products especially can accumulate these compounds, often quantified as antioxidant protection degree (214) or total antioxidant capacity (216). Unsaturated fatty acids, phenols, and volatile compounds are likewise transferred from forages to dairy products and play important roles in taste and odor as well as eventual consumer health (217). These are incredibly rich in grazing systems, at times ten times greater than in stall-fed ruminant diets (218). Therefore, vitamin and mineral supplementation often becomes the best management option only in confined feeding operations or monoculture grazing systems.

### Importance

The α-tocopherol and related compounds (vitamin E) and closely associated selenium (Se) are common feed-related deficiencies in confined ruminants not fed fresh green forages (219). Both are important in antioxidation processes that mitigate stress. Vitamin E, in conjunction with Se, plays a crucial role in cellular antioxidation. When deficient, physiological and immunological functions can be impaired, as can growth performance in confinement (220).

Retinol (vitamin A) is fat-soluble and plays an important role in ruminant eyesight, bone development, epithelial cell function, reproduction, as well as general immune functions (221). In ruminants, retinol enhances antioxidation that protects against cellular free-radicals (222). Carotenes are retinol precursors, and, under pasture or rangeland conditions, over 10 carotenoids have been documented in forages that can meet ruminant requirements (221). Their presence in milk produces distinctive butter and cheese colors that consumers identify with grazing-based dairy. However, feeding trials in confined feeding systems where fresh, green forage was lacking indicate that retinol supplementation to sheep (223) and calves (224) increases its presence in animal tissue, indicating that deficiencies may occur. There is also evidence that Vitamin A can interfere with Vitamin E retention in ruminant blood plasma, liver, and fat tissue.

Ascorbic acid (vitamin C) inhibits cortisol release, is a robust cellular antioxidant, and plays a vital role in ruminant products' fatty acid profile, especially dairy (225). Its supplementation to confined ewes, for example, increases the antioxidant concentration in milk (226). It also affects lamb, but not kid, meat quality parameters when administered before transport and slaughter (227). Diet can be a strong determinant of herbivore blood and milk ascorbic acid concentrations (228, 229), and its injection in confined cattle can reduce mortality rates (230).

Folic acid and vitamin B_12_ supplemented to confined multiparous (older) dairy cows can reduce dystocia by 50% and speed up first breeding postpartum by 3.8 days (231). It has no effect on primiparous dairy cows or any other health or reproductive factor for either class of animals. This indicates that, in confined feeding conditions, these can be essential supplements in multiparous ruminants where vitamin B can become depleted over time. No similar positive effect of folic acid and vitamin B_12_ supplement in pastured ruminants has been observed.

#### The Ruminant Animal's Perspective

Stress on ruminants affects animal health by increasing cellular oxidation. Stresses include abiotic factors, such as climate (mainly temperature extremes) or management, including transport or handling (219). Biotic stresses include interaction with other animals, reproduction, lactation, and feed quantity and quality deficiencies, as well as numerous other potential interactions with the living environment. Oxidative stress occurs when reactive oxygen species or free radicals surpasses the detoxification capacity of antioxidants. Activation of inflammatory-immune response and decreased overall immune function can result. There is evidence indicating that oxidative stress during weaning and transport plays a crucial role in the occurrence of bovine respiratory disease (232, 233) and affects feed efficiency (234) in newly received feedlot cattle. Ingesting antioxidants, such as vitamin E and related Se, can help reverse these adverse effects. When these are limited in the diet of confined ruminants consuming a limited diversity of fresh forages, supplementation can mitigate the adverse effects of stress on growth and product quality (226, 235, 236).

#### The Ruminal Microorganisms' Perspective

Ruminal microbes can synthesize as well as degrade vitamins and other antioxidative dietary compounds. Diet affects this dynamic. High energy concentrate diets, for example, result in an 80% vitamin A loss in the rumen compared to only 20% breakdown in high-forage diets (237). As a result, slow-release rumen boli containing vitamins and minerals have proven effective for enhancing confined ewe reproductive functions (238). However, it is unclear if vitamins played any role and their effectiveness declines after the initial weeks. The effectiveness of slow-release Cu, Se, or Co has proven especially useful in pastures where soils and consequently forages are low in any one of these minerals. However, because forages typically supply vitamins above rumen microorganism requirements, their supplementation has not been widely studied in grazing or browsing ruminants. In a feedlot where fresh forages are rarely an ingredient, however, this picture changes drastically.

#### The Consumers' Perspective

Volatile compounds ingested by grazing and browsing lactating ruminants change milk and dairy product fatty acid profile and antioxidant properties (239–241). Not only can this extend product shelf life, but it can also be important for health benefits to consumers as well as unique flavors in milk, butter, and cheese that arise from consuming certain forages that vary by region and season (214). These are driven by forage composition, particularly dicotyledonous plant species (242). When animals are fed in confinement, supplementation can compensate for vitamin deficiencies in the animal, which is then reflected in the product (235). In North American milk production, where strong flavors are not a consumer preference, forages containing these compounds may not always be desirable.

### Sources of Vitamins

The role of Vitamin E and other antioxidants in ruminant nutrition and health has been well-documented. Without them, animal health suffers, and production yield and quality decline. What is not always recognized is that their supplementation is largely irrelevant to pasture or rangeland-fed animals that ingest these naturally from fresh, green forages. These antioxidants readily appear in products originating from these free-ranging ruminants (214). Grazing and browsing ruminants, especially in ecosystems with diverse plant species, rarely benefit from dietary supplements. The same is not the case for confined feeding operations or monoculture grazing systems.

Confined animal feeding operations for feeding ruminants high energy diets invariably enhance animal production and health when they include synthetic vitamins and other antioxidant-enhancing supplements in the feed. This will come from fresh green forages or, in their absence, as synthetic supplements. These are generally injected to increase efficiency and bypass rumen degradation, but slow-release ruminal boli may also play a role in systems that do not lend themselves to repeated injections (238).

Very little is known about the antioxidant efficacy of feeding conserved (e.g., hays and silages) vs. freshly harvested (greenchop) forages to confined ruminants. Feeding trials comparing cut-and-carry or greenchop systems to conserved forages should also examine the role of forage species, functional groups (e.g., legumes), plant maturity, environment (e.g., soil nutrients or moisture), browse vs. grazing (especially for goats), and species diversity. Additional trials should examine the benefits of allowing animals to graze, browse, or even pen-feed selectivity (self-medication) for forages that lend themselves to greater antioxidant activity in the ruminant, animal products, and humans who consume products containing high or low concentrations of ruminant-originating antioxidants. Additional research should compare the efficacy of plant vs. synthetic vitamin sources in ruminant diets.

Should vitamins be systematically quantified in ruminant feedlot diet components? Quantifying vitamins important in ruminant cellular antioxidant functions in confined animal feed may not be as useful as measuring key minerals, mostly because the former is broken down by rumen microorganisms fed high concentrate and high energy feeds, making these unavailable for absorption in the remainder of the gastrointestinal tract. Supplementing vitamins up to minimum recommended levels has already been proven beneficial to ruminants in confinement, under heavy reproduction pressure, or under management-induced stresses, such as handling or transport.

### Future Perspectives

Additional research topics needing attention include the effectiveness of slow-release rumen boli for vitamins in feedlot systems. Timing (reproduction, weaning, season, maturity), rumen microorganism breakdown leading to inefficiencies, and duration of release all merit attention. The efficacy of slow-release supplements for confined feeding vis-à-vis fresh forages (classes, species, maturity, diversity) also merits focus, especially regarding animal and human consumer health benefits.

The key question is, should we invest resources in this phytochemical? For pasture-based systems that include diverse forage species, including legumes and other forbs, any investment is unlikely to produce any measurable benefit except in cases where soils are deficient in key minerals, such as Se, important for antioxidant health. More research is needed in the case of confined feeding operations, especially long-duration systems, such as confined dairies. Examples include comparing the economic and health returns of year-round fresh, diverse forage systems where mild climates allow cultivation during any season.

## Alkaloids

### Classification and Definitions

Alkaloids represent the largest class of secondary plant compounds in North-American perennial plants and occur in many rangeland grasses and weeds (243), where they mostly have gained attention as a potential toxin for ruminants and other pasture livestock in case of overfeeding of alkaloid-containing plants. Alkaloids were initially classified as cyclic compounds containing N in a negative oxidation state, derived from an amino acid. However, some pseudo-alkaloids are not derived from amino acids and alkaloid-like compounds (amines) that do not contain N within any ring-structure. Given the confusing nomenclature of alkaloids, pseudo-alkaloids, and amines, it seems more convenient to classify them based on their biogenetic origins, where four groups were created: (1) alkaloids derived from ornithine, arginine, lysine, histidine, phenylalanine, tyrosine, tryptophan, anthranilic acid, and nicotinic acid; (2) purine alkaloids (e.g., xanthine caffeine); (3) aminated terpenes (e.g., diterpene aconitine, triterpene solanine); and (4) polyketide alkaloids (e.g., coniine, coccinellines) (39). Alkaloids may be produced by plants and fungi infesting certain pastureland plants, such as the endophytic fungus *N. coenophialum* in tall fescue that contains the alkaloids peramine, ergot, and loline (244).

### Nutritional Importance

Overall, forage plants that include significant concentrations of alkaloids are considered toxic as many adverse effects in livestock exist, including acute and chronic symptoms, such as damage to the central nervous system, liver damage, muscle cramps, and death (245). The toxicological effects associated with alkaloids, specifically the broadly present class of pyrrolizidines, has been in discussion since the 1960s, specifically in context with animal production (246). Specifically, breeding efforts to remove tannins from forage for ruminants to optimize meat production may have possibly reduced tannins and alkaloids' interactions, thus increasing the toxicity of the latter (247). Many plants with high alkaloids in the leaf are not palatable to herbivores due to bitterness (248). It has been observed that wild animals (e.g., deer, rabbits) tend to limit the consumption of alkaloid containing plants but also to be highly tolerant. This resistance to chronic alkaloid intoxication has been, in part, ascribed to intestinal microbiome containing strains that can degrade alkaloids (249).

Initial efforts to remove alkaloids from the food chain of livestock production did not consider the crucial role of alkaloids across several ecological networks (245). Ergot alkaloids (e.g., ergovaline, ergonovine, ergine) are commonly found in tall fescue (*Festuca arundinacea*—now *Schedonorus arundinaceus* Schreb.; https://plants.sc.egov.usda.gov), but an endophytic fungus—*Neotyphodium coenophialum*—produces them. Through a mutualistic symbiotic relationship, it enables the tall fescue to thrive during drought and cold weather and resist insect predation, nematode infestation, and some diseases (244), but it can be devastating to the ruminant animal (250, 251). In a previous study, a genetically modified non-producing-ergot *N. coenophialum* has been incorporated into tall fescue to still yield the plant's agronomic benefits without causing toxicity to the grazing animal (244). Similarly, perennial ryegrass (*Lolium perenne* L.), another widely used cool-season pasture grass, is infected with *N. lolli*—an endophyte fungus that produces the biologically active ergot, peramine, and lolitrem alkaloids, which cause ryegrass staggers in livestock (244). In contrast, reed canarygrass (*Phalaris arundinacea* L.) produces the alkaloid gramine in leaf sheaths and stems, reducing ruminant's forage intake, thus limiting growth and development (244).

Simultaneously, various therapeutic activities have been ascribed to alkaloids, including antioxidant, cancer-preventive, antidiabetic, anti-inflammatory, and vasodilatory activities (252–254), but it has not been well-investigated how livestock could benefit from these beneficial activities from alkaloids. Many plant extracts that have been investigated for the beneficial actions of contained polyphenols and terpenoids may also contain alkaloids contributing to their biological activities, for example, giant milkweed (255) or herbal mixtures containing polyphenols, terpenoids, and alkaloids (256).

Additionally, the microbiome of ruminants, including bacteria, archaea, protozoa, and fungi, in part, metabolizes alkaloids to non-toxic metabolites (257); however, causal relationships have not been well-investigated. For example, Koester et al. (258) showed that cows with high vs. low tolerance to fescue toxicosis have vastly different microbiota compositions, specifically fungal phylotypes Neocallimastigaceae, potent fiber-degrading fungi, were consistently more abundant in the tolerant cattle. Additionally, it has not been well-investigated, which microbial enzymes are required to perform alkaloid metabolism (259).

### Future Perspectives

Overall, alkaloids' beneficial role to ruminants and their synergistic contributions to ecological networks in forage-animal management has not been well-investigated. The contribution of alkaloids in complex plant extracts beneficial to ruminant nutrition also remains to be explored.

## Essential Oils

### Classification and Definitions

Unlike the previous phytochemicals that maintain a reasonably specific chemical makeup, EO are mixtures of compounds comprised of previously discussed phytochemicals and other intrinsic chemicals. Indeed, the nomenclature “essential oils” is a misnomer because EO is neither essential in the sense that animals have a daily requirement nor are oils because they contain glycerol (2). The term EO was likely derived from quinta essentia (i.e., quintessence) attributed to Bombastus Paracelsus von Hohenheim^1493−1543^, who used the term for any extraction of pharmacological drugs via steam distillation (260). Essential oils are classically defined as complex, multi-component mixtures of various volatile and non-volatile compounds, including acids, acetones, alcohols, aldehydes, esters, phenolics, and terpenes (261). The primary constituents of EO are low molecular weight terpenes/terpenoids and aromatic compounds, with monoterpenes representing 90% of EO (262). Essential oils are commonly extracted from materials found throughout the plant, including bark, leaves, flowers, roots, seeds, and stems. The biological properties of an EO are determined by its chemical profile that can vary depending upon the extraction process, plant material, plant maturity, and growing environment (262). In many cases, much of the pharmaceutical properties exhibited by EO can be attributed to the phytochemicals that comprise an EO (e.g., terpenes, terpenoids, phenolics, polyphenolics) (261).

Essential oils can exhibit antimicrobial, antiseptic, antiparasitic, antioxidant, anti-inflammatory, and immuno-modulating activities. In general, EO display hydrophobic or lipophilic attributes that result in a high affinity for bacterial cell membranes, generating ion leakage that can ultimately result in ATP depletion and cell lysis (263, 264). Since ancient times, EO have been exploited by humans for their pharmaceutical properties (263), with EO currently being used regularly in agriculture, cosmetic, food, homeopathic, pharmaceutical, and therapeutic industries (262). Essential oils are cited as improving animal health and nutritional status by stimulating the circulatory, digestive, and immune systems, as well as reducing pathogenic bacteria and parasites (261, 265).

### Nutritional Importance

The nutritional effects of EO are primarily attributed to their antimicrobial properties that are comprised of multiple interaction mechanisms. Gram-positive bacteria are considered more susceptible to EO than gram-negative bacteria due to both hydrophobic and lipophilic interactions affecting cell membrane stability (266). However, small molecular weight components, via hydrophobic interactions, may be able to penetrate and affect gram-negative bacteria (267). The application of EO in ruminant nutrition has focused on ruminal modulation to shift the microbial consortium toward one that improves nutrient use efficiency (268). Significant emphases have primarily remained focused on N-metabolism, CH_4_ abatement, and the VFA profile (36, 264). Essential oils' complex and varied composition may provide the potential to alleviate tolerance and resistance developments associated with medically important antimicrobials and synthetic compounds.

#### Ruminal Fermentation

The basis for employing EO in ruminant diets is to modify the microbial population so that efficient fermentation pathways are used, and the animal's nutrient use efficiency is increased. The primary means of accomplishing this is by altering the VFA profile (lower acetate-to-propionate ratio) and a reduction in fermentative waste products (e.g., CH_4_ and NH_3_). The mode of actions provided by EO suggests they may be able to modify ruminal fermentation similar to ionophores by decreasing the prevalence of Gram-positive bacteria, including hyper-ammonia producing bacteria and those that readily produce formate or H_2_ (269).

*In vivo* research has demonstrated that EO reduce the acetate-to-propionate ratio to a level comparable to ionophores when ruminants are fed high-quality diets (e.g., dairy and feedlot) (270–274). However, this result is inconsistent, and it is not easy to discern if the decreased acetate-to-propionate ratio results from reduced acetate, increased propionate, or both, as all scenarios have been observed. An increase in butyrate has also been indicated in some studies (270, 275) and is cited as an indication that EO and ionophores have differing modes of action (264, 270). As well, ruminal branched-chain volatile fatty acids have been reduced (270) and increased (276, 277) *in vivo*, indicating an alteration in the cellulolytic microbes or those that synthesize branched-chain volatile fatty acids from branched-chain amino acids. Both branched-chain volatile fatty acids and branched-chain amino acids are essential for the normal fermentative functions of cellulolytic microbes in the rumen (1). Overall, the addition of EO often imparts no change to the total VFA concentration (277, 278). However, increased (271, 279) and reduced (280, 281) total VFA concentrations have been reported, but the reduction in total VFA concentration is typically not to the extent observed with ionophores (2, 22, 282).

The effect of EO on digestibility is a significant point of contingency, but it has not been a focal point for much of the *in vivo* work in beef cattle. Of those that have examined digestibility, there was no effect on DM digestibility or neutral detergent fiber digestibility (271, 272, 283). The result is similar in dairy cattle, with only marginal effects on digestibility (274, 281, 284, 285). As with digestibility, EO's inclusion does not appear to affect significantly intake, at least not at the supplementation levels commonly used *in vivo*.

The provision of EO *in vivo* has not demonstrated a repeatable effect on ruminal CH_4_ without suppressing digestibility. Supplementing diets with EO has decreased CH_4_ in dairy cattle (286–289), but did not change of increased CH_4_ production in beef cattle (277, 278). Although CH_4_ production has not been measured, when feeding EO, protozoa and methanogen numbers decline *in vivo* with a corresponding reduction in the acetate-to-propionate ratio (270, 272). A reduction in CH_4_ without inhibiting digestion has typically been observed when EO are provided at ~500 mg/kg DM, but as little as 41 mg/kg DM has imparted an effect. The beneficial effects are thought to be due to selective inhibition of protozoa and methanogens; however, the negative or ineffectual results are likely the result of EO demonstrating indiscriminate binding or lack of adequate biological activity.

Much research has investigated the potential application of EO to reduce proteolysis and deamination in the rumen. However, the consensus indicates that EO have little-to-no effect on the ruminal breakdown of protein and amino acids in beef or dairy cattle. The vast majority of research indicates no difference in ruminal NH_3_ when EO are included in the diet (275, 278–280). Similarly, numerous studies have failed to indicate a difference in blood or milk urea N from animals provided EO (274, 290, 291). The lack of effect is thought to result from EO being supplemented at too low of a rate to alter N metabolism (264). However, reduced ruminal digestibility had no effect on ruminal NH_3_ or blood urea nitrogen levels in beef heifers supplemented with EO (292, 293). This could indicate that some species of hyper-ammonia-producing bacteria are less sensitive to EO (294).

#### Post-rumen Digestion

Essential oils increase the flow of non-microbial N to the small intestine, as well as stimulate digestive enzymes and alter microbial populations in the lower tract. However, minimal investigation of rumen outflow and post-rumen digestion has been performed, particularly *in vivo*. In beef heifers, a linear increase in the flow of non-microbial N to the duodenum has been observed with an increasing rate of eugenol or cinnamaldehyde (292, 293). However, post-ruminal N digestibility does not improve when feeding EO (292, 293, 295, 296). The inclusion of EO yields equal or lesser ruminal N digestibility with no difference in intestinal digestibility. This results in total-tract N digestibility not different or lower than the control. A similar trend is present for starch and neutral detergent fiber digestibility, ruminally and post-ruminally. However, increased total-tract acid detergent fiber digestibility has been observed and attributed to a stimulatory effect of EO on digestive enzymes (283, 291, 297). In ruminants, no research has directly investigated EO as a stimulus for gastric or intestinal enzymes. However, this is not implausible as EO have demonstrated the ability to reduce pathogenic fecal bacteria (298) and diarrhea in calves (299), as well as reduce fecal DM and viscosity in dairy cattle (274).

### Methodological Aspects

Essential oils have primarily been investigated using *in vitro* methods, batch or continuous culture, particularly when screening multiple compounds and rates. In many instances, batch incubations have not adequately represented the dynamic rumen environment, whereas continuous culture has provided fermentation and outflow data comparable to *in vivo* results. Over the past decade, *in vivo* methods have been regularly implemented, but efforts have mainly focused on dairy and feedlot sectors, with little to no investigation into grazing beef cattle. Much of the *in vivo* research focused on fermentation parameters and digestive functions has utilized low animal numbers (4–16) in Latin square or switchback designs. Some larger pen-fed studies have emphasized performance and carcass characteristics with digestive attributes being investigated with a small number of cannulated animals. A shortcoming of multiple fermentation studies is that the use of low animal numbers has not greatly progressed our knowledge of the inter-animal variation associated with EO provision. For *in vivo* investigations, the length of EO or treatment provision varies greatly. Research focusing on digestion and fermentation commonly utilized 14- to 31-day feeding periods, whereas the larger performance trials typically ranged from 80 to 205 days on feed. There is an apparent deficiency in fermentation and microbiota data for animals fed EO for more than 30 days, limiting our knowledge of digestive or microbial alterations with prolonged feeding.

### Research Data

The successful application of EO depends on numerous factors, but the overall effect of EO is unclear due to a lack of consistency among measured variables. Even so, EO have regularly increased intake and improved the VFA profile and feed conversion in feeder cattle, as well as reduced CH_4_ and increased milk yield and feed conversion in dairy cattle. The reason for the different effects between a feeder and dairy cattle is likely, at least in part, a result of differences in diet composition, particularly the level and type of roughage. However, there is little information to assist in making comparisons to high-roughage diets. In a meta-analysis of the essential oil blend, Agolin Ruminant®, in dairy cattle, Belanche et al. (300) determined that supplementation of the EO blend increased milk yield and reduced CH_4_, and an adaptation period of at least 4 weeks was required for consistent results. Unfortunately, most digestive studies have utilized periods spanning 3–4 weeks, perhaps not allowing enough time for consistent outcomes to be realized. In a meta-analysis investigating the effects of EO in sheep diets, it was determined that EO increased neutral detergent fiber digestibility and propionate concentration and reduced protozoa populations and acetate concentrations (301). However, in contrast to dairy cattle, EO efficacy in sheep appeared to be highest within the first 30 days and then began diminishing. Regardless of species, the methodologies commonly used to study fermentation have not progressed our knowledge of EO efficacy with prolonged feeding.

### Future Perspectives

There appears to be potential for EO to improve animal efficiency and performance, but the variation among studies makes it difficult to parse out the effect of EO vs. random variation. If EO are to be commercially used in ruminant production, emphasis should be placed on using methods that improve the consistency of results (i.e., increased replication, extended feeding periods, recovery methods, chromatographic methods to determine purity). Research using forage diets merit increased attention, as improved fermentation profiles would greatly benefit this sector. Apart from nutrition, there seem to be EO opportunities for internal and external parasite control. Multiple *in vitro* studies have reported acaricidal activity of EO and have successfully used them to control cattle ticks (302–304), with evidence indicating EO as a potential method of controlling flies and lice (305–307). This is a vital area of research due to the rapid increase in parasite resistance to synthetic compounds, providing a large opportunity to investigate feed-through and topically administered EO in ruminant species. Another area that merits attention is the effect of EO on thermal stress. There is scientific and anecdotal evidence indicating that EO's provision may reduce the stress associated with hyperthermia (308–310), but the underlying mechanisms and efficacy in a production scenario are unknown. Although EO's nutritional effects may not be consistent, there is potential for EO to improve other health parameters that directly and indirectly affect the nutritional status of ruminants.

Should researchers invest resources in EO? Based on the current literature, adequate data point to the benefits of EO to ruminant production. Additional efforts should invest in the long-term and diversity of these compounds. However, research projects must be performed in a manner that better capture the effect of EO and promotes consistency among trials, rather than focusing on the least publishable unit.

## Conclusion

Many scientists embarked on alternative replacements to antibiotics in animal operations in the last 15 years after widespread concern over AMR due to antibiotics' perceived broad use in animal production. Phytochemicals became the preferred research pursuit, even though these compounds have been studied and applied in many fields long before AMR became publicized. Phytochemicals embody a broad spectrum of chemical components produced by plants and some fungi to act as chemicals against predatorial microbes, insects, and herbivores. Therefore, the idea of using them to manipulate ruminal fermentation and to establish other phytochemoprophylactic (prevent animal diseases) and phytochemotherapeutic (treat animal diseases) activities gained sympathizers.

Flavonoids comprise only 9% of known phytochemical compounds, but most research has been dedicated to this group, especially CT. However, because of inconclusive or contradictory findings, more targeted research is needed to confirm and validate published findings before definitive recommendations of phytochemicals usage in ruminant nutrition are drawn, such as what, when, and how much to use. Although some discoveries are encouraging, disagreements and lack of repeatability exist among studies, particularly for CT and saponins.

Alkaloids may also have a potential untapped benefit in ruminant nutrition. Although humans have long used alkaloids for their pharmacological properties, their phytochemical usage as feed additives in ruminants has not been sufficiently scrutinized. In part, given the intricacies in measuring and classifying alkaloids chemically, they may act as ghost compounds alongside other phytochemicals of known importance as plants produce many phytochemicals concurrently.

Likewise, terpenes, vitamins, essential oils, and other natural plant antioxidants play a large role in rumen ecology and function. These are most prevalent but least studied in fresh forages, especially in rangelands. The difficulty of isolating their individual effects in forage-based systems make them especially challenging to describe. This, however, does not detract from the critical roles they plan in ruminant ecosystems. The importance and individual effects are more easily identified in feedlot situations where concentrates and preserved forages contain fewer compounds, with consequent adverse effects on rumen microbiome health and ruminant nutrition. More research in these compounds in concentrated animal feeding operations is therefore merited.

The phytochemicals' role in sustainable ruminant production is undeniable, but much uncertainty remains. Scientists have yet to answer the sustainability issues before relying exclusively on phytochemicals as a sensational remedy for AMR, especially in complete rations lacking fresh forages and precluding ruminant feed selection. Phytochemical feed additives may have a place in sustainable production scenarios only if more convincing results of their efficacy and effectiveness in replacing antibiotics are dependably identified. The old saying “do not put all your eggs in one basket” still applies to phytochemical research.

## Author Contributions

All authors listed have made a substantial, direct and intellectual contribution to the work, and approved it for publication.

## Conflict of Interest

The authors declare that the research was conducted in the absence of any commercial or financial relationships that could be construed as a potential conflict of interest.

## Abbreviations

AMR: antimicrobial resistance
BW: body weight
CPP: ciliate protozoa population
CT: condensed tannins
DM: dry matter
DMI: dry matter intake
EO: essential oils
GIN: gastrointestinal nematode
HT: hydrolyzable tannin
TPS: triterpenoid saponins
and VFA: volatile fatty acids.
